# Can a digital intervention ‘Momentum’ improve social functioning and transdiagnostic symptoms for Australian youth at ultrahigh risk for psychosis? Protocol for a superiority randomised controlled trial comparing treatment as usual with and without Momentum

**DOI:** 10.1136/bmjopen-2025-102132

**Published:** 2025-12-25

**Authors:** Shaminka N Mangelsdorf, Daniela Cagliarini, Barnaby Nelson, Carla McEnery, James Whiting, Gina Chinnery, Lee Valentine, Yong Yi Lee, Cathrine Mihalopoulos, Virginia Liu, Sarah Bendall, Peter Koval, Simon D’Alfonso, Cesar Gonzalez-Blanch, Richard M Ryan, Reeva Lederman, Michelle H Lim, Patrick McGorry, Andrea Polari, Alison Yung, David L Penn, Eoin Killackey, John Gleeson, Mario Alvarez-Jimenez

**Affiliations:** 1 Orygen, The National Centre of Excellence in Youth Mental Health, Parkville, Victoria, Australia; 2 Centre for Youth Mental Health, The University of Melbourne, Parkville, Victoria, Australia; 3 Health Economics Group, School of Public Health and Preventive Medicine, Monash University, Melbourne, Victoria, Australia; 4 School of Public Health, The University of Queensland, Herston, Queensland, Australia; 5 Queensland Centre for Mental Health Research, Wacol, Queensland, Australia; 6 School of Public Health and Preventive Medicine, Monash University, Clayton, Victoria, Australia; 7 Melbourne School of Psychological Sciences, The University of Melbourne, Parkville, Victoria, Australia; 8 KU Leuven Research Group of Quantitative Psychology and Individual Differences, Leuven, Flanders, Belgium; 9 School of Computing and Information Systems, Faculty of Engineering and IT, The University of Melbourne, Parkville, Victoria, Australia; 10 IDIVAL, University Hospital Marques de Valdecilla, Santander, Spain; 11 Institute for Positive Psychology and Education, Australian Catholic University, Fitzroy, Victoria, Australia; 12 College of Education, Ewha Womans University, Seoul, South Korea; 13 Prevention Research Collaboration, Sydney School of Public Health, Faculty of Medicine and Health, The University of Sydney, Sydney, New South Wales, Australia; 14 The Institute for Mental and Physical Health and Clinical Translation (IMPACT), School of Medicine, Deakin University, Geelong, Victoria, Australia; 15 School of Health Sciences, University of Manchester, Manchester, UK; 16 Department of Psychiatry, University of North Carolina at Chapel Hill, Chapel Hill, North Carolina, USA; 17 Healthy Brain and Mind Research Centre, School of Behavioural and Health Sciences, Australian Catholic University, Fitzroy, Victoria, Australia

**Keywords:** Digital Technology, Psychosocial Intervention, Randomized Controlled Trial, Schizophrenia & psychotic disorders, MENTAL HEALTH, Child & adolescent psychiatry

## Abstract

**Introduction:**

Existing psychological and pharmacological interventions for young people at ultra-high risk (UHR) for psychosis have shown benefit in at least delaying the transition to psychosis, but they have limited benefit for comorbid disorders or social dysfunction, which are prominent for those at UHR. We developed a moderated online social therapy platform (named Momentum) including: (1) transdiagnostic therapeutic interventions targeting social functioning, depression, generalised anxiety and social anxiety; (2) a moderated, peer-led online community and (3) specialised human support from clinicians, career consultants and peer workers. The aim of this trial is to determine whether, in addition to treatment as usual (TAU), Momentum, a 12-month digital intervention, informed by the complex intervention framework, is superior to 12 months of TAU in improving social functioning in UHR young people.

**Methods and analysis:**

The study design is a prospective, parallel group, rater-masked randomised controlled trial. We will recruit young people aged 14–27 years, meeting one or more UHR for psychosis criteria. Participants are randomly assigned to the condition using randomly permutated blocks with a 1:1 allocation ratio. Participants are stratified by age (<18 years and ≥18 years), sex at birth and recruitment site. A total of 220 young people will be recruited, allowing for an 18% attrition rate following randomisation. The study includes a 12-month treatment phase, with assessment points at baseline, and 4 months, 8 months and 12 months. The primary outcome is social functioning, as measured by the Global Functioning Social Scale. Secondary outcomes include the severity of depressive and anxiety symptoms, social anxiety, role functioning, study and employment outcomes and cost-effectiveness. We will also examine potential mechanisms to understand Momentum’s therapeutic impact on social functioning. Primary analyses will be undertaken on an intention-to-treat basis. Mixed-model repeated measures analyses will be used to compare change in social functioning between the two treatment groups over the 12-month follow-up for primary, secondary and exploratory outcomes.

**Ethics and dissemination:**

Melbourne Health Human Research Ethics Committee (HREC/42964/MH-2018) provided ethics approval for this study. Findings will be made available through scientific journals and forums and to the public via social media and the Orygen website. De-identified individual participant data will be available after publication for 3 years via the Health Data Australia catalogue (https://www.researchdata.edu.au/health). Requests must include a methodologically sound proposal. Specific conditions of use may apply and will be specified in a data sharing agreement (or similar) that the requester must agree to before access is granted. Supplementary material including study protocol, informed consent material and statistical analysis plan will also be available.

**Trial registration number:**

Australian New Zealand Clinical Trial Registry (ANZCTR), ACTRN12619001411134.

**STRENGTHS AND LIMITATIONS OF THIS STUDY**

- *Momentum* is the first intervention to harness scalable digital technology with transdiagnostic content and multidisciplinary care to address social functioning in young people at ultra-high risk for psychosis.
- *Momentum* was developed by a multidisciplinary team in partnership with young people with lived experience and clinicians, which may enhance the acceptability of the intervention.
- The purpose of *Momentum* is to scale across, and embed within, early psychosis and youth mental health services.
- Due to the nature of psychosocial interventions, participants and moderators were not masked to treatment allocation.

## Introduction

### Background and rationale

 Late adolescence and early adulthood are key developmental periods, where social and emotional functioning broadens.^1^ It is during this critical period that psychotic disorders most commonly have their onset, often following a disabling and relapsing course, which results in enormous human suffering and societal costs.^2 3^ Earlier onset of psychotic symptoms is associated with worse and more treatment-resistant psychosis later in life.^3^ Therefore, early intervention is recognised as the best approach to prevent or delay the first episode of psychosis.^2 4^ Ultra-high risk (UHR) criteria (also known as Clinical High Risk) were developed to identify those at high risk of developing a psychotic disorder, including individuals with subthreshold symptoms and/or a family history of psychosis.^5^ Around 25% of those meeting UHR criteria transition to psychosis within a 3-year period.^6^

More recently, it has been suggested that an early risk state such as UHR may presage transition to more than one type of disorder, including but not limited to psychotic, mood and personality disorders.^7^,^9^ Regardless of the transition to new onset disorders, on average 78% of young people with UHR meet criteria for comorbid psychiatric disorders, most commonly major depressive disorder (30%), anxiety disorders (34%) and social anxiety disorder (14%).^10^ Difficulties in social functioning are also common among UHR young people, including fewer meaningful and satisfying relationships, marked loneliness and social isolation.^11 12^ Social functioning difficulties persist regardless of transition to psychosis and predict transition to psychosis^11 13^ and poor general functioning.^14^ Conversely, social functioning indicators, like employment and higher socioeconomic status, are protective factors against transition to psychosis.^15 16^

Existing psychological and pharmacological interventions have been found to be of benefit in at least delaying the transition to psychosis. However, they do not show superiority over treatment as usual (TAU) for comorbid disorders and social dysfunction.^17^,^19^ For example, cognitive-behavioural therapy for psychosis primarily targets transition from UHR to psychosis, without showing benefits for comorbidity or functioning.^18 20^ Transdiagnostic approaches specifically targeting comorbidity and social functioning are therefore recommended to address the heterogenous clinical presentations in the UHR population.^9 18 20 21^

UHR interventions have also overwhelmingly been delivered face-to-face.^18^ Digital mental health interventions (DMHIs) may enhance existing face-to-face treatments through blended care.^22 23^ DMHIs have been shown to be acceptable, engaging and feasible for use by individuals with psychosis and serve to harness the increasing availability and use of personal digital devices by individuals with psychosis.^24^,^27^ Furthermore, DMHIs may offer a unique opportunity to flexibly and concurrently address the needs of UHR young people through targeting social functioning as well as comorbid anxiety and depression.

*Momentum* is a DMHI informed by the complex intervention framework^28^ and based on the Moderated Online Social Therapy (MOST) model, designed to work alongside existing face-to-face services.^29^,^34^ To pre-emptively address difficulties with engagement for this population,^35^ *Momentum* has been designed with young people and incorporates evidence-based human support strategies to support engagement derived from self-determination theory (SDT)^36 37^ and persuasive design principles (described below).^38^

As a complex DMHI,^28^ *Momentum* consists of several interacting components with multiple concurrent treatment targets: (1) transdiagnostic content addressing one or more of social functioning in the context of UHR, depression, generalised anxiety and/or social anxiety; (2) three types of human support addressing (a) engagement with therapeutic content through clinical support, (b) functional recovery through specialised career support and (c) community engagement through peer support and (3) an online community feed moderated by peer workers. In an uncontrolled pilot study (n=14)^39^*, Momentum* demonstrated safety and acceptability in UHR young people, high usage and improvements in social functioning, which were significantly associated with the level of engagement. As in the case of other complex interventions, formal evaluation of *Momentum* in a randomised controlled trial (RCT) involves a broad range of outcomes.^28^ TAU was chosen as the comparator to *Momentum* as an adjunct to TAU, in order to test the real world implications of adding *Momentum* to existing care.

### Objectives

The aims of the *Momentum* study are to:

1. Evaluate, via an RCT, the effectiveness of *Momentum* in combination with TAU in improving social functioning (primary outcome), comorbid symptoms (depression, generalised anxiety and social anxiety), role functioning, study and employment outcomes in UHR young people (aged 14–27 years) compared with TAU alone over a 12-month period.
2. Evaluate the cost-effectiveness of adding *Momentum* to TAU.
3. Explore potential therapeutic mechanisms of *Momentum* (exploratory aim).

The primary hypothesis is that compared with TAU alone, TAU plus *Momentum* will lead to improved social functioning outcomes in UHR young people. The secondary hypotheses are that compared with TAU, TAU plus *Momentum* will reduce the severity of depressive, anxiety and social anxiety symptoms, improve role functioning, improve study and employment outcomes and be cost-effective (as defined via a cost-utility analysis whereby quality-adjusted life years (QALYs) will be used as the main measure of outcome).

## Methods and analysis

### Patient and public involvement

The *Momentum* intervention has been co-designed with young people, following participatory design principles^40^ with continual feedback from young people across the development, pilot and intervention period. Consistent with best practice in developing novel interventions,^41^ we obtained feedback from participants in the *Momentum* pilot^39^ that the intervention would benefit from more accessible and visually engaging therapy content. As a result, we incorporated graphic narratives and comics to enhance engagement with therapy content in *Momentum*. Participants in the intervention group will be invited to regular focus groups with the emphasis being on feedback and questions about the intervention. More broadly, the protocol and participant information and consent forms have been reviewed by the Orygen Youth Research Council^42^ and the investigator group carefully considered the burden of the trial schedule of assessments on participants.

### Trial design and setting

The study design is a prospective, parallel-group, rater-masked, superiority RCT with a 1:1 allocation ratio. Approximately 220 participants at UHR for psychosis will be allocated to either TAU or TAU + *Momentum*.

The trial includes a 4-year recruitment period, commencing in November 2019, and a 12-month treatment phase, with the study being completed within 5 years (December 2024). The design comprises four monthly assessment points across 12 months. The protocol development addressed all aspects of Good Clinical Practice,^43^ Consolidated Standards of Reporting Trials EHEALTH criteria^44^ and Standard Protocol Items: Recommendations for Interventional Trials guidelines.^45^

Participant recruitment commenced in October 2019 at services in the North-Western Melbourne (Victoria, Australia) catchment. Specifically, recruitment took place through the Personal Assessment and Crisis Evaluation (PACE) programme, a part of the Orygen Specialist Program and headspace centres led by Orygen (in the Melbourne suburbs of Sunshine and Glenroy). These services support young people who may be at risk of developing psychosis.^46 47^ Orygen is the world’s largest research and knowledge translation organisation focused on early intervention in mental ill-health in young people.

Orygen Digital, the digital mental health division of Orygen and Centre for Youth Mental Health at The University of Melbourne, designs, delivers and evaluates evidence-based digital services for youth mental health. In April 2020, Orygen Digital commenced implementation of the MOST platform across all Victorian youth mental health services both as part of the Victorian State Government’s response to the COVID-19 pandemic and in response to the recommendations of the Royal Commission into Victoria’s Mental Health System.^48^ Due to the overlap between study and implementation sites, participant recruitment from Victorian sites ceased in October 2020. Instead, satellite recruitment sites were established at two services within the South-Eastern Sydney Local Health District (Bondi Junction Community Mental Health Centre and headspace Bondi Junction) and at headspace services in the Illawarra region, New South Wales (NSW), operated by Grand Pacific Health (Wollongong, Nowra).

### Eligibility criteria

Inclusion criteria for participants are: (a) age 14–27 years inclusive; (b) able to read and converse in English; (c) able to provide informed consent; (d) able and willing to nominate an emergency contact person, such as a close family member; (e) meeting criteria for one or more UHR for psychosis groups.^49^

Exclusion criteria are: (a) past history of a psychotic episode of 1 week or longer; (b) acute risk of self-harm requiring urgent intervention (ie, suicidal ideation with a current plan and intent to enact this plan) at time of screening assessment; (c) attenuated psychotic symptoms only present during acute intoxication; (d) organic brain disease known to cause psychotic symptoms, for example, temporal lobe epilepsy; (e) any metabolic, endocrine or other physical illness with known neuropsychiatric consequences (eg, thyroid disease); (f) diagnosis of a serious developmental disorder (eg, severe autism spectrum disorder); (g) a diagnosed permanent developmental delay or intellectual disability; (h) previous exposure to a MOST platform.

### Intervention and comparator

#### Comparator

The comparator will be TAU. TAU consists of a range of treatment options delivered by the treating youth mental health service during the episode of care.^46 47^ These can include individual or group psychotherapy, medication, care co-ordination, career support and drug and alcohol treatment. It also includes any generic medical or mental health services typically available to young people in the absence of enrolment in the study. These can include follow-up by a general practitioner, private psychiatrist, primary care youth mental health services or adult mental health services, which deliver multidisciplinary psychiatric care (including medical follow-up, case management and acute psychiatric care as appropriate). TAU as the control condition has been selected because: (1) TAU is the typical comparison condition in clinical trials evaluating the initial effectiveness of a novel intervention^50^; (2) the high standard of care at the treating services and (3) social functioning, the primary outcome variable, is resistant to current treatment approaches.^51^ Thus, given the novelty of the intervention, the high quality of TAU and the treatment-resistant nature of the primary outcome, use of TAU as the control condition is scientifically reasonable. Participants in both groups may access any service throughout the study period. Service use will be captured at each assessment point.

#### Intervention

*Momentum* is a DMHI informed by the complex intervention framework^28^ and based on the MOST model.^29 31 39 52^ Young people are encouraged to engage with clinical support, therapeutic content, peer work, career consultants and the online community. Each component of *Momentum* is outlined below.

#### Therapeutic content

In accordance with SDT, the therapeutic content is structured to foster intrinsic motivation by supporting autonomy (eg, offering choice and flexibility in navigating content), competence (eg, skills and strategy building) and relatedness (eg, fostering peer connection via ability to contribute and view other young peoples’ reflections). The therapy content is organised into *journeys*, each addressing a specific presenting problem, made up of *tracks* (akin to modules) that target the maintaining factors of that issue. These tracks feature multimodal *activities* designed to align with gold-standard intervention strategies, while also accommodating different learning styles and ensuring accessibility for diverse users.

**Figure 1:**
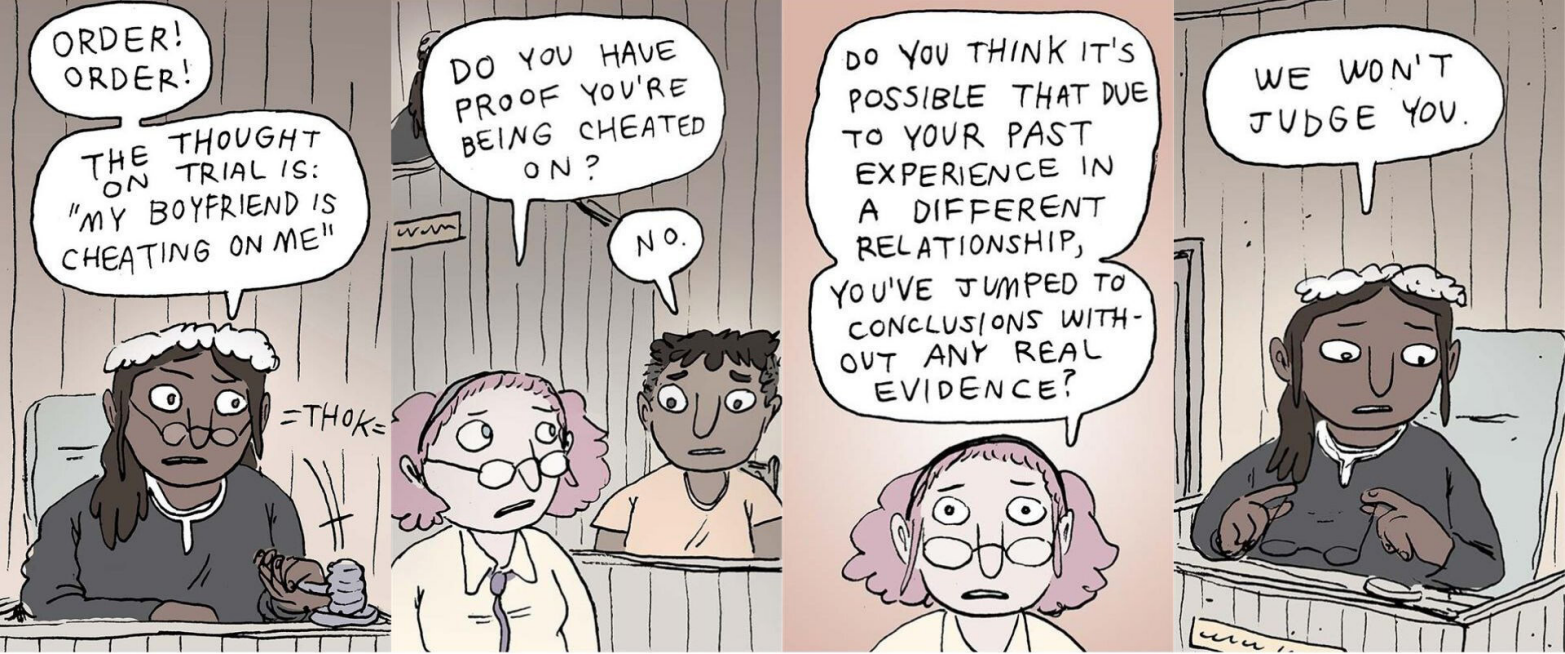
Four comic panels taken from one of the therapy comics in the Social Hacks journey titled ‘Thoughts on Trial’. The comic illustrates cognitive disputation as an intervention strategy via a narratively driven comic based within a court setting.

The five activity types include: *comics, reflective actions, actions, talking points* and *learning pages*. The comics, built on graphic medicine principles,^53^ use illustrated, multipanelled narratives with recurring characters to bring therapeutic concepts to life (see figure 1 for an excerpt). Reflective actions provide clear prompts designed to promote self-awareness and insight, while actions suggest practical steps, functioning as behavioural experiments to apply previously learnt skills and strategies in the real world. Talking points encourage social interactions by allowing users to view and comment on others’ reflections and experiences. Finally, learning pages summarise each track and offer psychoeducation. Users can save activities to a personalised toolkit, creating a labelled, accessible bank of strategies for future use.

*Momentum* offers five distinct therapeutic ‘journeys’ addressing social functioning, comorbid symptoms and vocational recovery, each designed using evidence-based frameworks and incorporating gold-standard intervention strategies targeting specific mental health challenges. These journeys include: enhancing social functioning (‘Social Hacks’), managing depression (‘Improve Your Mood’), managing anxiety (‘Find Your Calm’), overcoming social anxiety (‘Find Your Confidence’) and managing insomnia (‘Improve Your Sleep’). Additionally, there is a journey tailored to address work and study-related challenges. Users can access one journey at a time while also engaging with on-demand ‘Explore’ activities that complement their selected journey.

Participants randomised to the TAU + *Momentum* condition are initially offered the Social Hacks journey, which is underpinned by transdiagnostic tracks specifically aligned with mechanisms purported to enhance social functioning in young people. These include targeting self-defeating beliefs, enhancing motivation, strengthening mentalising capabilities, improving social cognition and fostering value-based goal setting.^54 55^ The Social Hacks journey contains over 60+ activities, available to use over the 12 months of participation in the trial. The platform is specifically designed to offer flexibility to the user in terms of how often they log in and complete content. However, young people are suggested to log in for around 2–10 min per day in order to complete an activity per day. It should take around 3 months to complete the Improve Your Mood journey at this rate, and young people have the opportunity to complete other journeys of choice across the 18-month intervention period. Intervention strategies in this journey include behavioural activation, value-based actions, cognitive restructuring, mindful awareness, effective interpersonal skills, self-compassion and psychoeducation on key mental health concepts.

#### Peer-to-peer online social networking (‘the Community’)

The ‘Community’ is a newsfeed-style moderated online space, designed to facilitate social support and enhance relatedness on the platform. Young people and peer workers (see below) can post about their lived and living experience of mental ill-health using text, emojis, images and links. Peer workers may also post icebreaker-type posts and career consultants post about work and study issues monthly. Users may ‘react’ (eg, ‘I get you’, ‘thinking of you’) or comment in response to a post.

All users (staff and young people) develop a profile listing their interests; however, all communication between young people is viewable by the community rather than in private messages. The Community has easily accessible guidelines for use to protect the safety of participants. Users can also use the ‘vent’ function to allow certain offensive words (most commonly swear words) that would otherwise be blocked by the safety system. Vent posts are collapsed in the feed by default so that their viewing is optional, and other users who wish to see such posts are required to click expand to view them. Allowing young people to vent in a safe way on the Community provides an immediate, judgement-free outlet for authentic emotional expression, helping to reduce stress and prevent the buildup of negative emotions, particularly for those with low social supports.^56^

The Community also includes Talk it Out (TiO), a structured problem-solving function informed by the social problem-solving framework.^57^ TiO provides an opportunity for the young person to co-develop solutions with the Community. TiO steps a young person through three phases of (1) brainstorming the emotional impact and facts of the situation, (2) redefining their concern as a problem question (eg,‘How can I manage stress?’) and (3) supporting the young person to visualise how they might feel if their problem was solved. TiO provides evidence-based problem-solving tips and examples throughout and checks in with the young person after receiving feedback from the Community. TiO is moderated by peer workers, who can also provide assistance on drafts before the post is made to the Community.

#### Human support

The *Momentum* platform is moderated by clinicians, peer workers and career consultants. *Momentum* moderators work together as a multidisciplinary team, meeting fortnightly as a whole team to review engagement on the platform and discuss engagement strategies (often influenced by SDT). Moderators can also communicate with one another via chat messaging and discipline-specific activity logs on *Momentum*. The moderation team is led by the trial moderation coordinator who is also a clinician. Each discipline has a specific moderation manual that guides processes during the trial intervention period (available on request).

*Clinicians*: each young person on *Momentum* receives support from a mental health clinician (eg, clinical psychologist, social worker) with experience working with young people. The clinician is a supportive and trusted expert, providing support in line with the supportive accountability model.^58^ Contacts may be synchronous (ie, phone or video calls) or asynchronous (ie, SMS/chat message). *Momentum* clinicians also ensure clinical safety on the platform by monitoring the clinical status of young people in their caseload (through all synchronous and asynchronous contact and reviewing research measures) and conducting safety checks two times per day of all written text by young people on the platform.

Clinicians welcome their allocated young people to the platform via an initial ‘welcome call’, during which they provide a platform orientation, support the young person to set goals for their time on the platform (eg, how often to complete content) and develop a formulation. The platform orientation is typically performed via video call, while all other synchronous interactions are via phone unless offering a video call may assist in boosting engagement with the platform. The formulation then informs SDT-informed support provided (appropriately balancing autonomy, competence and relatedness) and the selection and tailoring of the therapy journey. Clinicians aim to maintain weekly contact with the young person across the 12-month intervention period.

The clinician’s primary aims are to support engagement and to suggest and tailor therapy content. While the Social Hacks journey is the first journey offered to young people during the trial, users may also choose to switch to a different journey in consultation with their allocated clinician. In determining which journey to focus on, clinicians will consider the young person’s preference, as well as the young person’s most prominent presenting concern (ie, depression, anxiety, social anxiety or social functioning) based on their onboarding/ongoing assessment scores and formulation. The clinician may also tailor the journey according to the transdiagnostic therapy targets (ie, most essential content; see table 1) of the journey to sustain engagement while maximising therapeutic effectiveness.

**Table 1:** Therapy targets for each journey

Journey name	Social Hacks	Improve Your Mood	Find Your Calm	Find Your Confidence
Target	Social functioning	Depressive relapse	Generalised anxiety	Social anxiety
Essential therapy targets	Addressing cognitive distortions (in a social context) Social skills and acting with confidence Initiating pleasant and novel activities and committing to valued social action Compassion for self and others	Addressing rumination Addressing cognitive distortions Self-compassion Mindful awareness	Mindful awareness Relaxation Addressing cognitive distortions Self-compassion Managing avoidance	Excessive self-awareness Addressing cognitive distortions (ie, shame, perfectionism) Managing avoidance and safety behaviours

Clinicians may also suggest, and young people can access, on-demand ‘explore’ activities to sustain engagement or content from the Improve Your Sleep journey, as relevant. As other forms of human support are optional, the clinician may also proactively suggest engagement with peer workers and/or career consultants.

In order to strengthen fidelity to the moderation model and manualised processes, clinicians attend weekly peer supervision sessions led by the trial moderation coordinator and complete bi-monthly fidelity checklists to discuss and address their self-identified strengths and weaknesses.

#### Career consultants

Career consultants provide specialised education and vocational support including career exploration, planning and transition, managing uncertainty and indecision, guidance on education pathways and enrolment, study support, job application processes, career assessments and discussions around disclosure and reasonable adjustments. Career consultants on *Momentum* are experienced and qualified in career development practice and registered with national regulatory bodies. Functional and vocational outcomes, including return to usual career-related activities (eg, work, school, further education), and building vocational skills that can provide purpose, improve self-confidence, self-efficacy and career adaptability. These skills can further improve vocational outcomes and resilience to career shocks which affect ongoing mental health, recovery and relapse prevention.

Career support is available to all young people on *Momentum*. Career consultants work flexibly, responding to individual needs to establish key goals. Support is provided through direct messaging, with phone contact offered depending on needs and preferences. Career consultants consider all potential influences on a young person’s career including the environment and perceived self-efficacy, thus working within the Systems Theory Framework of Career Development^59^ and social cognitive career theory.^60^ The overarching approach ensures consistency with the key theoretical underpinnings of *Momentum,* including SDT and supportive accountability to build engagement, trust and encourage completion of activities.

Career consultants draw on a repertoire of evidence-based career education activities within *Momentum,* aligned to the Australian Blueprint for Career Development,^61^ and direct young people to relevant and reputable external resources where required. All content and engagement aims to build employability skills aligned to the Core Skills for Work Developmental Framework^62^ and guided by career construction theory,^63^ supporting the young person to undertake activities to build their own career self-management skills and career adaptability. A collaborative approach with peer workers and clinicians on *Momentum* and relevant external stakeholders supports each young person to work towards their education and vocational goals.

#### Peer workers

Peer workers are young people who have been trained to intentionally share their lived and living experience of mental ill-health. Peer workers moderate the online ‘Community’ (including TiO) and chat individually with young people on the platform via direct messaging. Peer workers also lead monthly online ‘hangouts’ to reinforce connections and promote engagement. Peer workers are supervised by the peer work coordinator. The peer work model is designed to normalise experiences, counteract stigma and promote platform engagement. Peer workers are thus integral to the provision of social support via *Momentum*—a proposed mechanism of action in the study.^64^

### Persuasive systems design framework

The principles in the Persuasive Systems Design (PSD)^38^ framework are intended to influence users’ behaviours and attitudes, guiding them toward achieving specific goals. These principles are aimed at enhancing user motivation, increasing engagement and promoting long-term behaviour change, making systems both effective and compelling in achieving desired outcomes.

*Momentum* incorporates 14 of the 28 principles outlined in the PSD. Table 2 provides a detailed overview, including the domain, principle, description and an example of how each persuasive principle is applied within the intervention.

**Table 2:** Application of persuasive design principles in the *Momentum* intervention

Principle	Description	Momentum example
Reduction	Reducing complex behaviours into manageable tasks	Therapeutic activities are segmented into bite-sized steps. Clinicians support young people to set manageable goals for platform use.
Tunnelling	Guiding the user through a process or experience in a manageable way	The system guides young people through their therapeutic journey, providing a clear and structured pathway for progression.
Tailoring	Customising information to reflect the needs, interest, personality and usage context of a user group	Customised content and activities align with young people’s developmental stage and specific mental health needs.
Personalisation	Providing individualised content and experiences	Personalised content recommendations that are linked to the young person’s specific goals.
Rehearsal	Providing opportunities to practise a behaviour to help users prepare for real-world situations	Encourages young people to practise behaviours by applying them in real-world scenarios, reinforcing learning through repetition and real-life application.
Praise	Providing praise or positive feedback for using the system	Moderators acknowledge progress and reinforce positive behaviour.
Reminders	Remind users of their target behaviour	Clinicians can send reminders, ensuring timely prompts to encourage engagement and task completion.
Suggestions	Offer relevant suggestions to the user	Clinicians suggest relevant content, offering tailored recommendations that support progress and engagement.
Liking	A visually attractive system	Designed in combination with young people and designers to create user-friendly and visually appealing experience.
Surface credibility	The system conveys a competent appearance	Provides clear and accessible information, such as terms and conditions, contact phone numbers and distress support advice.
Trustworthiness	Information provided should be fair and objective	Ensures content is supported by evidence-based theories and practices.
Expertise	Information provided should demonstrate knowledge, experience and competence	*Momentum’s* content is designed by a team of clinicians, and the moderation team is staffed by psychologists, social workers, peer support workers and career consultants, ensuring expertise and professional oversight in the platform’s delivery.
Normative influence	Facilitate the gathering of users with similar goals, fostering a sense of community and shared norms	The community within *Momentum* fosters a sense of belonging by bringing together young people with similar lived experience. This creates a supportive environment that promotes normalisation and encourages social connections, reinforcing the notion that young people are not alone in their journey.
Social facilitation	Users can perceive that others on the system are performing the target behaviour	*Momentum* supports social facilitation by providing talking points on content, allowing young people to discuss and share their experiences of trying new skills.

### Safety protocol

The safety protocol comprises three levels of security: (1) system and privacy protection; (2) online safety and (3) clinical safety.

The *Momentum* platform is hosted on Amazon Web Services. Amazon Web Services and Orygen Digital meet standards of responsible business practice. Identity management and networking are handled by Orygen Digital’s engineering department according to Orygen (National) Standards, which meet Australian research requirements. In addition, *Momentum* has a wide range of measures to secure the application and database against unauthorised access. These measures conform to industry best practice as defined by the Open Web Application Security Project (www.OWASP.org). Privacy and online safety are managed in accordance with the Australian Communications and Media Authority.

On onboarding, the *Momentum* clinician carries out an initial orientation with *Momentum* participants, including details of the terms of use. Participants are required to accept and comply with the guidelines for safe use of *Momentum*. When needed, participants are offered guidance on appropriate usage of the system. All users are asked to nominate an emergency contact person, such as a close family member. *Momentum* includes a ‘report function’ that enables young people to report a concern about any material posted by a user. Moderators assess each report and respond accordingly, which may include removing material and, in some cases, deactivating or restricting the post author’s account. Participants are also able to hide their profile and activity should they become concerned about their privacy.

Clinical risk is managed through both manual and automated procedures. First, clinical moderators monitor the system twice daily on weekdays and once daily on weekends for evidence of clinical risk or deterioration. Any detected increased risk activates the *Momentum* risk and safety protocol, which includes one or more of the following: a risk assessment with the young person, informing the research team, alerting the emergency contact nominated by the participant and liaising with suitable emergency services where necessary. In addition, the system incorporates visible emergency guidelines and contact information. Finally, *Momentum* includes an automated keyword detection function, which activates each time a participant posts a contribution indicative of clinical risk, or a post containing potentially offensive words. The function blocks posts with notifications sent to the young person and the moderator, who can ‘unblock’ the post should they determine it unproblematic.

If, while in contact with a young person, the clinical moderator becomes aware of a clinically significant deterioration of symptoms, increased risk of suicide or a hospital admission, the clinician performs an assessment to determine the risks and benefits of a temporary withdrawal from *Momentum*. Based on this assessment, and in consultation with the young person, the clinical moderation team determines whether their account is temporarily suspended, or their level of access is restricted. Following suspensions or restrictions to a user’s account, the clinician will contact the young person at monthly intervals to ascertain whether the account is to be reactivated.

### Outcomes

#### Primary outcome and measure

The primary outcome is social functioning over 12 months. Social functioning will be measured by the Global Functioning: Social Scale, a valid and reliable instrument designed to measure social functioning in UHR.^65 66^

#### Secondary outcomes and measures

Secondary outcomes and measures include:

1. *Depressive* symptoms will be measured by the Quick Inventory of Depressive Symptomatology-Self-Report self-report instrument^67^ at baseline, 4 months, 8 months and 12 months.
2. *Anxiety* will be measured by the Generalized Anxiety Disorder 7-item Scale^68^ at baseline, 4 months, 8 months and 12 months.
3. *Role functioning* will be measured using the Global Functioning: Role Scale.^65^
4. *Vocational and education status* will be self-reported by participants at baseline, 4 months, 8 months and 12 months (including the participant’s report of employment and educational activities in between assessments).
5. *Cost-effectiveness* will be assessed using an adapted self-reported Resource Use Questionnaire (RUQ) to determine the broader resource use of participants (eg, community mental health services, hospitalisations, work and educational impacts, etc) and the Assessment of Quality of Life-Eight Dimension (AQoL-8D) questionnaire, which measures health-related quality of life and can be used to calculate QALYs.^69^

#### Other measured outcomes

Additional exploratory outcomes, potential covariates and intervention-only outcomes are outlined in table 3.

**Table 3:** Schedule of enrolment, assessments and intervention

	Enrolment		Post randomisation		
	-t_1_ - 0	0	t1 (4 months)	t2 (8 months)	t3 (12 months)
Informed consent	✓				
Eligibility screen	✓				
Primary outcome and measure					
Social functioning (Global Functioning: Social)	✓		✓	✓	✓
Secondary outcomes and measures					
Depression (QIDS)	✓		✓	✓	✓
Anxiety (GAD-7)	✓		✓	✓	✓
Social anxiety (LSAS)	✓		✓	✓	✓
Role functioning (Global Functioning: Role)	✓		✓	✓	✓
Vocational status (RUQ)	✓		✓	✓	✓
Cost-effectiveness (RUQ and AQoL-8D)	✓		✓	✓	✓
Exploratory outcomes and measures					
Self-efficacy (SES)	✓		✓	✓	✓
Social support (SPS)	✓		✓	✓	✓
Subjective well-being (SWLS)	✓		✓	✓	✓
Transition to psychosis (Abbreviated CAARMS)	✓		✓	✓	✓
Attenuated psychotic symptoms (Abbreviated CAARMS)	✓		✓	✓	✓
Quality of life (AQoL-8D)	✓		✓	✓	✓
Stress (PSS)	✓		✓	✓	✓
Loneliness (UCLA Loneliness Scale^77^)	✓		✓	✓	✓
Self-esteem (Rosenberg SES)	✓		✓	✓	✓
Psychological well-being (BPNS)	✓		✓	✓	✓
Sleep quality (PSQI)	✓		✓	✓	✓
Suicidal ideation and attempts (CSSRS)	✓		✓	✓	✓
Strengths use (SUS)	✓		✓	✓	✓
Mindfulness skills (FMI)	✓		✓	✓	✓
Self-compassion (SCS-SF)	✓		✓	✓	✓
Substance use (ASSIST)*	✓				✓
Anomalous self-experience (IPASE)*	✓				
History of trauma (ACE)*	✓				
Randomisation		✓			
Intervention		Continuous			
Comparator		Continuous			
Digital phenotype (AWARE-Light^78^)		Continuous			
Daily social functioning and subjective well-being (SEMA3 application)		Daily			
Intervention group-only outcomes and measures					
Intervention acceptability (qualitative evaluation via semistructured interview)					✓
Therapeutic alliance (WAI-C, WAI-T)		✓	✓	✓	✓
Usage data		Continuous			

* Potential covariates.

ACE, Adverse Childhood Experience Questionnaire^79^; AQoL-8D, Assessment of Quality of Life – Eight Dimension^69^; ASSIST, Alcohol, Smoking and Substance Involvement Screening Test^80^; BPNSS, Basic Psychological Needs Satisfaction Scale^81^; BPNSS, Basic Psychological Needs Satisfaction Scale^81^; CAARMS, Comprehensive Assessment of At Risk Mental States^82^; CSSRS, Columbia-Suicide Severity Rating Scale^83^; FMI, Freiburg Mindfulness Inventory^84^; GAD-7, Generalized Anxiety Disorder 7-item scale^68^; IPASE, Inventory of Psychotic-Like Anomalous Self-Experiences^85^; LSAS, Liebowitz Social Anxiety Scale^86^; PSQI, Pittsburg Sleep Quality Index^87^; PSS, Perceived Stress Scale^88^; QIDS-SR, Quick Inventory of Depressive Symptomatology-Self-Report^67^; Rosenberg Self-Esteem Scale, Rosenberg SES^89^; RUQ, Resource Use Questionnaire; SCS-SF, Self-Compassion Scale Short Form^90^; SEMA3, Smartphone Ecological Momentary Assessment^91^; SES, Self-Efficacy Scale^92^; SPS, Social Provisions Scale^93^; SUS, Strengths Use Scale^94^; SWLS, Satisfaction with Life Scale^95^; WAI-T, Working Alliance Inventory client version (WAI-C) and therapist version^96^.

### Data collection methods

Multiple methods will be employed to assess study outcomes. These include blind interviewer ratings, self-report and usage data from the *Momentum* system.

Research assessments will be administered either in person, via phone or telehealth platform. The research assistants (RA) will administer interviewer-rated measures verbally, documenting the responses in a hard or soft copy of the case report form (CRF). Self-report measures are delivered to the participant electronically via Orygen National’s Research Project Management System (RPMS). The initial assessment will take approximately 1.5–2 hours, with follow-up assessments expected to take approximately 1 hour.

Research assistants will be trained in the administration of all interviewer-administered measures. Eligibility for the study will be confirmed after consultation with the study coordinator. Follow-up assessments will be discussed in weekly review meetings.

Several strategies will be embedded to promote participant retention, including a flexible assessment window, accommodating availability of research assistants and honing research assistant interview skills to minimise time commitment at larger assessment points.

### Harms

In this study, any adverse event (AE) that meets the following criteria will be recorded:

- AEs that meet a grading severity of 3 or more as per the Common Terminology Criteria for Adverse Events (CTCAE) V5.
- AEs assessed as possibly or related either directly or indirectly to the trial intervention or trial procedures.

AEs that, in the opinion of the study team or participant, are considered reportable

A serious AE (SAE) is any untoward medical occurrence that at any administration level:

- Results in death.
- Is life-threatening.
  - Life-threatening in the definition of serious refers to an event in which the participant was at risk of death at the time of the event, it does not refer to an event which hypothetically might have caused death if it were more severe.
- Requires inpatient hospitalisation or prolongation of existing hospitalisation.
- Results in persistent or significant disability/incapacity.
- Is a congenital anomaly/birth defect.
- Is an important medical event that, although not immediately life-threatening or resulting in death or hospitalisation, based on appropriate medical and scientific judgement, may jeopardise the participant and/or require intervention to prevent one of the outcomes listed above.

AEs occurring after screening but prior to baseline will be recorded as medical history, unless possibly related to a study procedure, in which case they will be reported as an AE. AEs will be tracked until the AE resolves, stabilises or the subject is lost to follow-up.

AE and SAE data may be derived from a number of sources including the research assessment, interaction with study clinical team or reported to the study team by the treating service.

Any SAE occurring during the study, irrespective of the treatment received by the participant, must be reported to the sponsor within 24 hours of the investigator or designee becoming aware of the SAE regardless of the relationship to the intervention. SAEs which are expected to be due to the participant’s underlying illness are not required to be reported in an expedited manner. These events are still required to be recorded for participant safety, data recording and tracking purposes. The investigator should always provide an assessment of causality at the time of the initial report. A follow-up report should be completed when outstanding information becomes available, when there is a significant change in the event or when the event resolves. SAEs will be reported to the governing Human Research Ethics Committee (HREC) in accordance with HREC requirements.

### Participant timeline

Following informed consent, participants are enrolled in the trial and screened for eligibility. Once eligibility is established, participants complete the baseline assessment within a 1 month window. Participants are randomised after the completion of baseline measures. Participants in the intervention group receive access to *Momentum* after randomisation for the duration of the study period. All participants complete follow-up assessments at 4 months, 8 months and 12 months, respectively.

The participant timeline is outlined in table 3.

### Sample size

Sample size was determined by power analysis using G*Power 3. The primary outcome is the difference in social functioning at 12 months. Our pilot study found a very large effect size (d=1.83, p<0.001) improvement in social functioning from baseline to 2-month follow-up.^39^ This contrasts with follow-up and treatment studies in UHR patients, where social functioning has not improved over time.^51^ Thus, we conservatively assume a medium treatment effect (Cohen’s *d*=0.45), for which a total sample of 180 participants is required to achieve 85% power (alpha=0.05) or 128 participants to achieve 80% power (Cohen’s *d*=0.5). The same applies to all continuous secondary outcome measures (eg, depression, anxiety, social anxiety, role functioning). A total of 220 young people will be recruited, allowing for an 18% attrition rate following randomisation. This compares favourably with the attrition rates in the *Momentum* pilot (7%).

### Recruitment

Participants will be recruited from primary and specialist youth mental health services. In Victoria, study RAs attend weekly clinical review meetings at PACE and headspace services to identify eligible clients. In NSW, a study clinical liaison is appointed at satellite sites to actively facilitate recruitment at the site. The study liaison attends clinical review meetings, engages with treating clinicians and screens clinical files to identify potentially eligible clients. The study liaison introduces the trial to the client and obtains consent to share contact details with Orygen. Young people can also self-refer via the study recruitment page on the Orygen website.

### Randomisation: sequence generation, allocation concealment mechanism and implementation

The randomisation schedule is generated by a statistician independent of the study, programmed in the RPMS and not accessible by the study team. Participants are randomly assigned to the treatment condition via the RPMS using randomly permutated blocks with a 1:1 allocation ratio. Participants are stratified by age (<18 years and ≥18 years), sex at birth and recruitment site.

Once eligibility is established, participants complete the baseline assessment, after which participants are randomised by the research assistant via a secure online RPMS. The RPMS sends an automated email to the study coordinator and principal investigator, notifying them of the outcome of randomisation. The study coordinator informs each participant of their treatment allocation via SMS.

### Blinding

Research assistants will be blind to treatment allocation. To maintain blinding, all participants will be reminded not to discuss treatment with the research assistant at the follow-up assessment. When unblinding does occur, every effort will be made for future interviews with the participant to be conducted by a researcher who is still blind to treatment allocation. Success of the blind will be assessed by recording the RA’s judgement of treatment allocation for each participant at 4 months, 8 months and 12 months follow-up.

### Data management

The RPMS is used to manage all outcome data. The RPMS includes an electronic CRF (eCRF). The research assistants record participant-level data on an eCRF. These data are subsequently entered into the eCRF section of the RPMS. Self-report data are entered directly by the participant. The RPMS includes range restrictions to minimise data entry errors. The RPMS is accessed using a secure website and is stored on a secure server. It is designed to maintain the privacy and confidentiality of participant information and to ensure the integrity of the data. Access to RPMS is restricted to study personnel and the level of access is dependent on the person’s role. Data will be stored for a period of at least 25 years after the final publication arising from the study.

### Statistical methods

Primary analyses will be undertaken on an intention-to-treat basis. Mixed-model repeated measures (MMRM) analyses, including random intercepts to account for individual variability in baseline levels of social functioning, will be used to compare change in social functioning between the two treatment groups over the 12-month follow-up. MMRM is the analysis of choice because assumptions of traditional data analysis methods (eg, Analyis of Variance (ANOVA), logistic regression) may be violated, such as the assumption of homogeneity of regression across time points.^70^ Time (baseline, 4 months, 8 months and 12 months) will be the within-subjects factor and group (*Momentum* vs TAU) the between-subjects factor. Random intercepts account for individual differences in baseline levels of the outcome (eg, social functioning at the start of the study). However, individuals may also differ in how their outcomes change over time (eg, the rate or direction of improvement/decline in social functioning). Random slopes model this variability by allowing each participant to have their own trajectory (slope) over time. We will test whether including random slopes significantly improves model fit, but if the variance of slopes is negligible, a simpler model with only random intercepts will be adopted. MMRM will also be used to analyse change in the continuous secondary and exploratory outcomes over 12 months. Group differences in risk of transition to psychosis and vocational outcome will be tested using Fisher’s exact test, an alternative to the χ^2^ test more robust to small sample sizes.^71^ Time to transition to psychosis will be assessed by survival analysis (using either proportional hazard or accelerated life-time models).

Exploratory mechanisms of action (self-efficacy, social support and subjective well-being) analyses will be conducted using a multilevel structural equation modelling framework to assess mediation.^72^ Specifically, person-specific slopes representing change over time in the proposed mechanism-of-action variables (eg, self-efficacy) will be tested as mediators of the effect of treatment group (*Momentum* vs TAU) on social functioning.

Additional analyses will use multiple imputation to assess the robustness of the findings to the choice of method for handling missing data. Additional comparisons between treatment groups based on completer-only analyses will be conducted. Analyses will be undertaken in accordance with International Council for Harmonisation (ICH) 9 guidelines including a full analysis as well as per protocol set. The per protocol sample will be defined based on receiving a prespecified minimal exposure to the online intervention (ie, more than six logins over the 12-month intervention period).

The economic evaluation will comprise a cost-utility analysis, in addition to a cost-consequences analysis, comparing the incremental costs of the *Momentum* intervention (vs TAU) to a wide range of incremental study outcomes (eg, QALYs, social functioning, etc). Inclusion of the AQoL-8D questionnaire facilitates derivation of QALYs and enables a cost-utility analysis to be undertaken. A study-specific RUQ was adapted for this study from another RUQ frequently used in Australian mental health-related economic evaluations.^73^ The RUQ encompasses: community-based health service use; hospitalisations; accommodation services; medication and diagnostic tests; impacts on education and employment and other relevant services. Unit costs for resource use items will be sourced from nationally representative data sources, where available, with sensitivity analyses determining the impact of adopting alternative unit costs.^34^ The cost of delivering the *Momentum* platform will be estimated by microcosting data obtained from study-related financial records and intervention coordinators. Best practice within-trial economic evaluation methods will be adopted.^74^ The comparative cost-effectiveness of the *Momentum* platform (vs TAU) will be summarised using the incremental cost-effectiveness ratio metric (ie, cost per QALY gained). Statistical methods involving generalised linear models will be used to compare differences in costs and QALYs between groups. Bootstrapping will also be used to characterise uncertainty around the incremental cost-effectiveness ratio. A range of cost-effectiveness ratios outlined within the Productivity Commission Inquiry into Mental Health^75^ will be used to assess whether the *Momentum* platform is value for money. Briefly, a cost-effectiveness ratio of $A64 000/QALY will be used as the primary value-for-money criterion, with $A96 000/QALY deemed marginally cost-effective. If the *Momentum* platform is found to be effective, then a population-level budget impact analysis will be undertaken.

### Qualitative analysis

Following the completion of the intervention period, young people will be invited to participate in a semistructured qualitative interview to explore their experiences and perspectives of the Momentum intervention. All interviews will be audiorecorded and transcribed verbatim for analysis. Data will be analysed thematically, following the six-phase approach outlined by Braun and Clarke.^76^

Analysis of the primary, secondary and exploratory outcomes will be conducted by a data analyst working within Orygen Digital but independent of the study team. The economic evaluation will be conducted by the health economics investigators. The qualitative analysis will be conducted by the lead qualitative investigator.

### Data monitoring committee

The study was assessed as low risk by the sponsor and a trial management group will be established in place of a data monitoring committee.

Discontinuation of the study will be considered where:

- A participant attempts suicide or self-harms, and it is highly likely that the attempt is related to the *Momentum* intervention.
- There are repeated instances of participants notifying moderators about triggering or distressing content posted by other users.

Decisions about study discontinuation will be determined by the trial management group in consultation with the sponsor.

### Trial monitoring

The sponsor monitoring plan includes an initial monitoring visit early in the recruitment phase which will determine the ongoing monitoring schedule.

## Ethics and dissemination

### Research ethics approval

Melbourne Health HREC (HREC/42964/MH-2018) provided ethics approval for this study.

### Dissemination policy

Findings will be made available through scientific journals and forums and to the public via the trial registry, social media and the Orygen website within 6 months of the completion of data analysis.

### Protocol amendments

Changes to the protocol may be initiated by the investigator group. The sponsor is notified of the planned amendment. The amendment is reviewed and approved by the HREC. The trial registry is updated in line with the amendment once the amendment has been approved.

### Consent or assent

The process of obtaining consent is staged. The RA, trained in obtaining informed consent by the study coordinator, contacts potential participants via SMS and schedules a time to introduce the study. During the introductory call, the research assistant provides a detailed explanation about the trial and offers to answer initial questions. After the study introduction call, the participant is provided with the full plain language statement and a study summary document which outlines the salient study details. The participant is provided with the opportunity to review the documentation and consider their participation. Once the participant confirms their decision to participate, a consent call is scheduled. The research assistant reviews the plain language with the participant and answers any outstanding questions. All participants are required to provide informed, signed consent. Parental or legal guardian consent and participant assent is required for participants under 18 years of age.

### Confidentiality

Information collected in connection with this research project will remain confidential with some limits. Limits to confidentiality include where the participant reports serious risk of harm to themselves or others. The limits of confidentiality will be explained as part of the informed consent procedure.

Identifiable information collected as part of the administration of the study is stored in the RPMS and access is restricted to key study personnel. All psychometric data will be reidentified for the purpose of analysis. Psychometric data shared through a data repository will be de-identified. Reports to the study sponsor or ethics committee may include re-identifiable data as required.

### Ancillary and post-trial care

Ancillary and post-trial care is not planned for this trial as the intervention is low-risk and non-medical.

### Protocol and statistical analysis plan

The protocol will be available via the trial registry (ANZCTR). The statistical analysis plan is limited to the protocol.
