# Supplementary material for: Can a digital intervention ‘Momentum’ improve social functioning and transdiagnostic symptoms for Australian youth at ultrahigh risk for psychosis? Protocol for a superiority randomised controlled trial comparing treatment as usual with and without Momentum
Source: BMJ Open. 2025 Dec 25;15(12):e102132. doi: 10.1136/bmjopen-2025-102132 (PMC12742139; doi:10.1136/bmjopen-2025-102132)
Supplement: Supplementary file 1 [file bmjopen-2025-102132.reviewer_comments.pdf]

PEER REVIEW HISTORY

BMJ Open publishes all reviews undertaken for accepted manuscripts. Reviewers are asked to complete a checklist review form and are provided with free text boxes to elaborate on their assessment. These free text comments are reproduced below.

ARTICLE DETAILS

Title (Provisional)

Can a digital intervention “Momentum” improve social functioning and transdiagnostic symptoms for Australian youth at ultra-high risk for psychosis? Protocol for a superiority randomised controlled trial comparing treatment as usual with and without Momentum.

Authors

Cagliarini, Daniela; Mangelsdorf, Shaminka N.; Nelson, Barnaby; McEnery, Carla; Whiting, James; Chinery, Gina; Valentine, Lee; Lee, Yong Yi; Mihalopoulos, Cathrine; Liu, Virginia; Bendall, Sarah; Koval, Peter; D'Alfonso, Simon; Gonzalez-Blanch, Cesar; Ryan, Richard M; Lederman, Reeva; H Lim, Michelle; McGorry, Patrick; Polari, Andrea; Yung, Alison; Penn, David L.; Killackey, Eoin; Gleeson, John; Alvarez-Jimenez , Mario

---

VERSION 1 - REVIEW

**Reviewer**	**1**
**Name**	**Rosebrock, Laina**
**Affiliation**	**University of Oxford, Department of Experimental Psychology**
**Date**	**12-May-2025**
**COI**	**None**

This is an ambitious trial for an important and vulnerable patient group. Several points:

1: The abstract should include the total number of patients to be recruited to the study.

2: The main confusion in the paper is just about how the intervention looks and is used by patients. How long do each of the activities take? How long do you expect each of the journeys to take? How often do you expect people to log in? The authors mention access for 12 months but it's unclear exactly how the programme is meant to be structured over 12 months. I appreciate this may be variable but some data from the pilot study would be useful here.

3. A figure/graphic showing the way you expect users to engage with Momentum would also be useful as it is confusing from the paper at the moment (i.e. journeys vs tracks). Table 1 is helpful but it appears that it does not include all elements.

4. Can the authors explain the stratification rationale? Why by age and gender?

5. The authors mention consent happens before screening for eligibility - can this be elaborated? What would make someone ineligible after consent/during screening?

It also appears as though recruitment was due to end in March 2025 - why is the protocol only being published now? Have you recruited to target or is an extension required?

**Reviewer**	**2**
**Name**	**Sauvé, Geneviève**
**Affiliation**	**Université du Québec à Montréal**
**Date**	**22-May-2025**
**COI**	**None**

Thank you for the opportunity to review this manuscript. This protocol presents a randomized-controlled study to evaluate the effectiveness of the Momentum digital mental health intervention. The authors provide a good rationale for the need of the intervention and present their protocol clearly. I have noted only a few instances in which a little more details would be beneficial. These are presented below.

- Please clarify the list of variables that will be tested for mediation effects and are considered proposed mechanisms of action.

- Has the protocol been registered on an online registry (e.g., clinicaltrials.gov)? If yes, please indicate platform and registration number.

- P.16, line 3; please provide more details on how clinicians monitor the clinical status of young people in their caseload. Is the monitoring done with information from the platform or the case file? If the former, how is it different from the safety checks?

- P.18, line 15; when 'phone contact' is described, is the contact done exclusively by phone or could it be done via videoconference (e.g. Zoom, Teams, Facetime)?

- P.26, line 7-9; will the mixed-model analysis include random slope and/or intercept? If yes, please provide details on how this will be modeled.

- Please specify who will be responsible for conducting the analyses (e.g. researchers, external statistician).

- P29, line 29-31; authors mention that follow-up assessment would continue until March 2025. Does this mean that data collection is completed? If yes, please specify in the text and mention whether have analyses started?

VERSION 1 - AUTHOR RESPONSE

Reviewer: 1
Dr. Laina Rosebrock, University of Oxford

Comments to the Author:
This is an ambitious trial for an important and vulnerable patient group. Several points:

1: The abstract should include the total number of patients to be recruited to the study.

This has been added, according to the SPIRIT guidelines.

2: The main confusion in the paper is just about how the intervention looks and is used by patients. How long do each of the activities take? How long do you expect each of the journeys to take? How often do you expect people to log in? The authors mention access for 12 months but it's unclear exactly how the programme is meant to be structured over 12 months. I appreciate this may be variable but some data from the pilot study would be useful here.

Thank you for this point – the intervention is specifically designed to offer flexibility to the user in terms of how often they log in and complete content. However, we have added the following to the paper to clarify this, as well as a useful rule of thumb:

"The platform is specifically designed to offer flexibility to the user in terms of how often they log in and complete content. However, young people are suggested to log in for around 2-10 minutes per day in order to complete an activity per day."

3. A figure/graphic showing the way you expect users to engage with Momentum would also be useful as it is confusing from the paper at the moment (i.e. journeys vs tracks). Table 1 is helpful but it appears that it does not include all elements.

Thank you for this point – several summary sentences have been included/clarified to specify what is available for young people in the intervention group:

"Young people are encouraged to engage with clinical support, therapeutic content, peer work, career consultants and the online community."

"The therapy content is organized into *journeys*, each addressing a specific presenting problem, made up of *tracks* (akin to modules) that target the maintaining factors of that issue. These tracks feature multimodal *activities* designed to align with gold-standard intervention strategies, while also accommodating different learning styles and ensuring accessibility for diverse users."

4. Can the authors explain the stratification rationale? Why by age and gender?

Stratification by age and gender is to ensure the mix of users on the platform is representative of the whole sample.

5. The authors mention consent happens before screening for eligibility - can this be elaborated? What would make someone ineligible after consent/during screening?

Thank you. Although potential participants are prescreened prior to the study being introduced to them, formal screening does occur until after the participant has consented. Formal screening includes assessment of all inclusion and exclusion criteria. It is possible that the formal screening assessment will identify ineligibility as it is more comprehensive than the prescreen which is based on notes in a medical record or clinician impression.

It also appears as though recruitment was due to end in March 2025 - why is the protocol only being published now? Have you recruited to target or is an extension required?

Recruitment was finalised in March 2024 and follow-up finalised in April 2025. A total of 85 participants were recruited. The recruitment period spanned 4 years and included a 1 year extension. One of the main challenges for recruitment was the impact of the pandemic. The clinical services were recruiting from prioritised clinical care and found it challenging to accommodate research needs. Furthermore, we discontinued recruitment from our local services due to the implementation of the MOST platform as standard care withing those services. The shift to interstate recruitment sites held early promise, but we were unable to recruit this population of participants at those sites as expected.

The delay in the publication of the protocol is a reflection of those challenges. Our team also ran a concurrent study in Major Depressive Disorder. The protocol for that study was published last year and was prioritised as it as recruitment ended earlier.

Reviewer: 2
Dr. Geneviève Sauvé, Université du Québec à Montréal

Comments to the Author:
Thank you for the opportunity to review this manuscript. This protocol presents a randomized-controlled study to evaluate the effectiveness of the Momentum digital mental health intervention. The authors provide a good rationale for the need of the intervention and present their protocol clearly. I have noted only a few instances in which a little more details would be beneficial. These are presented below.

- Please clarify the list of variables that will be tested for mediation effects and are considered proposed mechanisms of action.

The prosed mechanisms of action are self-efficacy, social support and subjective wellbeing. These have been added to the Statistical methods section.

- Has the protocol been registered on an online registry (e.g., clinicaltrials.gov)? If yes, please indicate platform and registration number.

The trial has been registered. Trial Registry details:

Australian New Zealand Clinical Trials Registry (ANZCTR)

ACTRN12619001411134

Date registered: 15/10/2019

https://www.anzctr.org.au/Trial/Registration/TrialReview.aspx?id=376773&isReview=tru
e

- P.16, line 3; please provide more details on how clinicians monitor the clinical status of young people in their caseload. Is the monitoring done with information from the platform or the case file? If the former, how is it different from the safety checks?

Thank you for this point – "monitoring clinical status" simply refers to using clinical judgement in interactions. The following phrase has been included:

"*Momentum* clinicians also ensure clinical safety on the platform by monitoring the clinical status of young people in their caseload (through all synchronous and asynchronous contact and reviewing research measures) and conducting twice-daily safety checks of all written text by young people on the platform."

- P.18, line 15; when 'phone contact' is described, is the contact done exclusively by phone or could it be done via videoconference (e.g. Zoom, Teams, Facetime)?

We have added a statement to clarify this:

"The platform orientation is typically performed via video call, while all other synchronous interactions are via phone unless offering a video call may assist in boosting engagement with the platform."

- P.26, line 7-9; will the mixed-model analysis include random slope and/or intercept? If yes, please provide details on how this will be modeled.

I have confirmed that the mixed-model analysis will include random intercept but I am unable to provide further detail about how this will be modelled due to the availability of the data analyst.

- Please specify who will be responsible for conducting the analyses (e.g. researchers, external statistician).

The analysis will be conducted by a data analyst working within Orygen Digital but not part of the study team.

- P29, line 29-31; authors mention that follow-up assessment would continue until March 2025. Does this mean that data collection is completed? If yes, please specify in the text and mention whether have analyses started?

Data collection has been completed in April 2025. Data analysis in planned for the last quarter of 2025. This sentence sat within the Discussion which has been removed from the resubmission, inline with BMJ Open SPIRIT 2025 template so this is not mentioned in the text.

Reviewer: 1
Competing interests: Not applicable

Reviewer: 2
Competing interests: Not applicable

VERSION 2 - REVIEW

**Reviewer**	**1**
**Name**	**Rosebrock, Laina**
**Affiliation**	**University of Oxford, Department of Experimental Psychology**
**Date**	**16-Sep-2025**
**COI**	

The authors have sufficiently addressed most of the comments, except for more detailed information about the intervention. Although they add information about how often patients are suggested to log in per day, this doesn't discuss how long each of the journeys/tracks take nor does it describe the general structure of therapy over 12 months. Ie is it expected that users complete at least one journey? Can they return to previously covered content even after moving on to another journey? I think a few more sentences here would really help.

VERSION 2 - AUTHOR RESPONSE

Reviewer: 1

Dr. Laina Rosebrock, University of Oxford

Comments to the Author:

The authors have sufficiently addressed most of the comments, except for more detailed information about the intervention. Although they add information about how often patients are suggested to log in per day, this doesn't discuss how long each of the journeys/tracks take nor does it describe the general structure of therapy over 12 months. Ie is it expected that users complete at least one journey? Can they return to previously covered content even after moving on to another journey? I think a few more sentences here would really help.

Authors: We have added further detail about journey completion.

Reviewer: 1

Competing interests: Not applicable

Previous Revisions

Dear Mrs. Cagliarini,

Following review of your article to BMJ Open, we invite you to submit a major revision.

The review comments can be found at the end of this email, together with any comments from the Editorial Office regarding formatting changes or additional information required to meet the journal's policies at this time.

Please note that your revision may be subject to further review and that this initial decision does not guarantee acceptance.

To submit your revised article please click this link: *** PLEASE NOTE: This is a two-step process. After clicking on the link, you will be directed to a webpage to confirm. ***

https://mc.manuscriptcentral.com/bmjopen?URL_MASK=8e7f00f15f404ed9ad9b06dd08121ffd. Alternatively, you can log on to your Author Dashboard in ScholarOne and under "Action" click "create a revision".

Please read and respond to all of the peer review comments. You should provide a point-by-point response to explain any changes you have (or have not) made to the original article and be as specific as possible in your responses.

The original files will be available to you when you start your revision. Please delete any files that you intend to replace with updated versions and upload the following using the appropriate file designation:
- “Main Document’’ - This is a clean copy (without tracked or highlighted changes) of your revised article. Please delete your original submission file.
- “Main Document - marked copy” - This is the edited version of your original article, including edits to address the peer review comments. Any changes have been highlighted using a track change function or bold or coloured text.
Please replace any other files that have been updated e.g. Images, forms.

The reviewers' comments, your response, and the previous versions of your article will be published as supplementary information alongside the final version of your article.
